# Features of Heart Failure with Preserved Ejection Fraction in Patients with Chronic Obstructive Pulmonary Disease and Systemic Sclerosis-Associated Interstitial Lung Diseases

**DOI:** 10.3390/jpm15050206

**Published:** 2025-05-20

**Authors:** Lyazat Ibrayeva, Meruyert Aubakirova, Irina Bacheva, Assel Alina, Nazira Bazarova, Aizhan Zhanabayeva, Olga Avdiyenko, Seda Borchashvili, Saltanat Tazhikhanova, Askhat Murzabaeyev

**Affiliations:** 1 Department of Internal Medicine, Karaganda Medical University, Karaganda 100012, Kazakhstan; libraeva@qmu.kz (L.I.); bacheva@qmu.kz (I.B.); alina@qmu.kz (A.A.); zhanabaevaa@qmu.kz (A.Z.); borchashvilis@qmu.kz (S.B.); tazhihanova@qmu.kz (S.T.); murzabaev@qmu.kz (A.M.); 2 Administration Department, Regional Clinical Hospital of Karaganda, Karaganda 100000, Kazakhstan; bazarovazakirova@mail.ru; 3 Scientific Research Laboratory, Karaganda Medical University, Karaganda 100012, Kazakhstan; avdienko@qmu.kz

**Keywords:** heart failure, systemic sclerosis, interstitial lung disease, chronic obstructive pulmonary disease

## Abstract

**Background/Objectives**: This study aims to investigate the potential etiopathogenesis of HFpEF development and identify possible different phenotypes of HFpEF in patients with chronic obstructive pulmonary disease (COPD) and systemic sclerosis-associated interstitial lung diseases (SS-ILDs). It could help clinicians improve early HFpEF personalized detection and management. **Methods**: This study included 150 patients with chronic lung diseases (CLDs), such as COPD and SS-ILD, who were outside of exacerbation, had no history of chronic heart failure (CHF), and had a left ventricular ejection fraction (LV EF) of ≥50%. The functional status of the lungs, heart, endothelial dysfunction, and acid–base balance was assessed. The results obtained were compared in groups of patients with CLD depending on the presence or absence of HF with preserved ejection fraction (HFpEF). The diagnosis of HFpEF was established based on the HFA-PEFF Score classification. Nonparametric statistical methods were used. **Results**: In patients with CLD, indicators such as age, longitudinal size of the right atrium, mid-regional pro-atrial natriuretic peptide (MR-proANP), and highly sensitive cardiac troponin T (hsTnT) were higher than in the group of patients without HFpEF. In patients with COPD and HFpEF, statistically significant changes were found in the volume of the left atrium. In patients with SS-ILD and HFpEF, statistically significant differenceswere found in SBP before and after the 6 min walk test (6MWT), the Borg scale before 6MWT, MR-proANP, and the longitudinal dimension of the right atrium. **Conclusions**: The results of our study allow us to identify two different mechanisms of HFpEF development: In patients with COPD, the predominant factor in the development of HFpEF was hypoxia, while in patients with SS-ILD, myocardial dysfunction with remodeling developed against the background of secondary pulmonary hypertension, highlighting the importance of phenotype-specific evaluation. These findings suggest potential approaches for personalized risk stratification and the development of targeted management strategies for patients with HFpEF.

## 1. Introduction

The study of chronic heart failure (CHF) development mechanisms in patients with chronic lung diseases (CLDs) is a pressing issue due to the nosological heterogeneity of CLD, the absence of unified registries, and the limited number of studies in this area. These factors hinder the development of effective approaches for the timely diagnosis and treatment of CHF in CLD.

Most modern studies focus on CHF with reduced ejection fraction (HFrEF) due to its poor prognosis. However, some research indicates that the outcomes of HF with preserved ejection fraction (HFpEF) are comparable to those of HFrEF [1,2]. Among CLDs, most data on CHF prevalence are available for chronic obstructive pulmonary disease (COPD): According to the Chinese registry, CHF prevalence is 11.6% [3], while in European and American populations, it reaches up to 25% [4]. CHF in COPD patients leads to a decline in quality of life, increased mortality, and a higher financial burden on healthcare systems worldwide [5]. Unrecognized CHF in COPD, and vice versa, contributes to a 50% one-year mortality rate [6]. The presence of HFpEF in COPD patients increases hospitalization rates by 1.54 times; cardiovascular mortality by 1.42 times; and all-cause mortality by 1.52 times [7], whereas, in HFrEF, comorbidity and hospitalization rates from all causes are even higher [2].

The pathophysiological mechanisms linking COPD and CHF form a complex network of interactions, necessitating an integrated approach to diagnosis, treatment, and management. The presence of one condition significantly affects the prognosis and clinical course of the other. CHF symptoms in COPD patients are often masked by respiratory failure and bronchial obstruction. Both conditions share common risk factors and pathogenic mechanisms, leading to delayed diagnosis and treatment, which reduces the effectiveness of medical interventions [8]. The cardiovascular complications observed in COPD suggest not only the coexistence of two distinct diseases but also an interaction within the cardiorespiratory continuum. Thus, COPD is not merely a comorbidity but an active participant in CHF development and progression.

Assessing the prevalence of CHF in interstitial lung diseases (ILDs) is challenging, as this category includes over 300 distinct nosologies with varying pathophysiological mechanisms. The available literature provides limited data on CHF prevalence in idiopathic pulmonary fibrosis (IPF), estimated between 11% and 20%. In systemic sclerosis-associated ILD (SS-ILD), HFpEF has been identified in 27% of patients [9]. However, no data are available on the prevalence, diagnosis, or prognostic impact of HFpEF in ILD.

Overall, the data suggest that HFpEF in chronic lung disease (CLD) is often masked by symptoms of the underlying condition and may carry a prognosis as serious as that of HFrEF. This prognosis appears to depend on personalized mechanisms of disease development. It can be assumed that among patients with CLD, there are distinct phenotypes of HFpEF based on etiopathogenetic mechanisms, clinical presentation, and laboratory findings. Identifying these phenotypes may help physicians enhance the early, personalized detection and treatment of HFpEF. Study objective: The objective of this study is to investigate the potential etiopathogenesis of HFpEF development and identify different possible phenotypes of HFpEF in patients with COPD and SS-ILD.

## 2. Materials and Methods

### 2.1. Study Population

This longitudinal study included 150 patients with chronic lung diseases (CLDs), such as COPD and SS-ILD, in a stable phase. All patients had a left ventricular ejection fraction (LVEF) of ≥50% and no history of chronic heart failure (CHF). Comorbid conditions among the patients with HFpEF included arterial hypertension (28 patients, 22%), chronic renal failure (4 patients, 3%), and atrial fibrillation (0 patients). Among the patients without HFpEF, arterial hypertension was present in 4 cases (20%), and chronic renal failure was present in 3 cases (15%). Patients were recruited at the outpatient clinic of Karaganda Regional Clinical Hospital between January and December 2023. The inclusion criteria were as follows: patients aged 25 to 60 years with CLD and no history of CHF. The exclusion criteria were as follows: pregnancy; severe chronic kidney disease (eGFR < 60 mL/min/1.73 m^2^, calculated using the CKD-EPI 2011 formula); acute conditions or diseases; and oncological and hematological diseases.

The study protocol included echocardiography, capnography, spirometry, arterial blood gas analysis, and laboratory tests (amino-terminal pro-B-type natriuretic peptide (NT-proBNP), mid-regional pro-atrial natriuretic peptide (MR-proANP), highly sensitive cardiac troponin T (hsTnT), endothelin-1, and galectin-3).

### 2.2. Clinical and Anthropometric Measurements

Anthropometric measurements were obtained using standard methods, and the body mass index (BMI) was calculated as kg/m^2^. The 6 min walk test (6MWT) was performed according to the recommendations of the American Thoracic Society [10]. Dyspnea severity was assessed using the Borg scale.

Quality of life was evaluated with the World Health Organization Quality of Life (WHOQOL-BREF) questionnaire. This short version of the WHOQOL questionnaire consists of 26 items covering four domains: physical health, psychological health, social relationships, and environmental factors.

For COPD patients, the disease’s impact was assessed using the COPD Assessment Test (CAT).

### 2.3. Laboratory Studies

Laboratory analyses were performed in two facilities: the research laboratory of Karaganda Medical University and the clinical diagnostic laboratory Olimp.

In the research laboratory of Karaganda Medical University, the blood levels of endothelin-1, hsTnT, and MR-proANP were measured using ELISA kits: Endothelin-1 (EDN1)—Cloud-Clone Corp. (Katy, TX, USA); High-sensitivity Troponin T (hsTnT)—Cloud-Clone Corp. (Katy, TX, USA); and Midregional Pro-Atrial Natriuretic Peptide (MR-proANP)—Cusabio (Houston, TX, USA). Analyses were performed on the EVOLIS IFA-robotic system (Bio-Rad Laboratories, Inc., Hercules, CA, USA). In the Olimp clinical diagnostic laboratory, NT-proBNP and galectin-3 levels were measured using the Alinity automated immunochemical analyzer (Abbott, Abbott Park, IL, USA).

### 2.4. Echocardiography

Echocardiographic measurements were performed using a VIVID IQ equipped with a 1.3–4.0 MHz (Wuxi, Jiangsu Province, China) 3Sc-RS phased array transducer according to the current recommendations of the American Society of Echocardiography/European Association of Cardiovascular Imaging. Early diastolic (E) and atrial (A) wave velocities and E-wave deceleration time were measured using pulsed-wave Doppler recordings from the apical four-chamber view. The early diastolic velocity of spectral pulsed-wave tissue Doppler (e′) was obtained by averaging the septal and lateral mitral annular velocities, and E/e′ was calculated to obtain an estimate of left ventricular (LV) filling pressure. Left atrial volume was measured using the biplane Simpson method from the apical two- and four-chamber views.

### 2.5. Capnography

Capnography was conducted using a portable capnograph/pulse oximeter Capnostream 35 (manufacturer Covidien llc., Jerusalem, Israel). The device delivers real-time, continuous monitoring of the patient’s respiratory status by measuring etCO_2_, SpO_2_, respiration rates, and pulse rates. A separate personal O_2_/CO_2_ Nasal FilterLine (Oridion Medical 1987 Ltd. ,Jerusalem, Israel) Adult cannula was used for each patient.

### 2.6. Spirography

Spirometry was used to measure the forced vital capacity (FVC) and forced expiratory volume in one second (FEV1). All tests were performed in accordance with the recommendations of the European Respiratory Society. The device used was BTL-08 Spiro Pro (Ashford, Kent, UK).

### 2.7. Statistical Analysis

Statistical analyses were performed using the SPSS version 22 software package (IBM, Armonk, NY, USA). The values were presented as medians with the values of the first and third quartiles (Me [Q1; Q3]). A total of 150 measurements of clinical, laboratory, instrumental studies, and questionnaires were analyzed. The normality of the distribution of variables was checked based on the Kolmogorov–Smirnov criterion.

Clinical, laboratory, and instrumental parameters were compared in groups of patients with chronic lung diseases depending on the presence or absence of HFpEF. The diagnosis of HFpEF was established based on the HFA-PEFF Score classification: With a score of more than 5, the result was interpreted as heart failure with preserved ejection fraction, and with a score of less than 5, the result was interpreted as the absence of heart failure [11]. Further differences were searched for depending on the presence or absence of HFpEF separately in groups of patients with COPD and SS-ILD. The Mann–Whitney U test was used to assess intergroup differences. In all statistical tests, *p* values below 0.05 were considered statistically significant.

## 3. Results

### 3.1. Study Group

The study group consisted of 150 patients (80 men—53%; 70 women—47%) with CLD, and the average age was 53.17 ± 8.75. There were 74 patients with COPD (65 men—88%; 9 women—12%) whose average age was 55.43 ± 6.83 years. Patients with SS-ILD numbered 76 (61 women—80%; 15 men—20%), and their average age was 50.96 ± 9.83 years.

### 3.2. Analysis of Differences Between Groups

The personalized characteristics of patients with CLD and with/without HFpEF are presented in Table 1. Patients with CLD and HFpEF were older by an average of 4 years (*p* = 0.041) and had a larger longitudinal right atrium (RA) (*p* = 0.027), and MR-proANP and hsTnT values were increased by 1.5–2 times (*p* = 0.01 and *p* = 0.015).

Table 2 presents the personalized results of the examinations of patients with COPD depending on the presence or absence of HFpEF. In patients with COPD, statistically significant differences of 1.8 times were found in the volume of the left atrium (*p* = 0.02). The complete version of the table is provided in the Supplementary Materials.

Table 3 presents the personalized results of examinations in the groups of patients with SS-ILD depending on the presence or absence of HFpEF. In patients with SS-ILD, statistically significant differences were found in the following criteria: SBP before and after the 6 min walk test (*p* = 0.04 and *p* = 0.006), Borg scale before the 6 min walk test (*p* = 0.008), MR-proANP level (*p* = 0.006), and longitudinal dimension of the right atrium (*p* = 0.005). The complete version of the table is provided in the Supplementary Materials.

Table 4 presents the personalized characteristics of patients with HFpEF in COPD and SS-ILD. Weight (*p* = 0.033) and respiratory rate (RR) before 6MWT (*p* = 0.034) in patients with COPD were higher than in patients with SS-ILD. The external respiratory function parameters were as follows: (FVC (*p* = 0.044), FEV1 (*p* = 0.007), FVC/FEV1 (*p* = 0.003), MEF 25 (*p* = 0.004), MEF 50 (*p* = 0.007), MEF 75 (*p* = 0.068), endothelin-1 (*p* = 0.030), and MR-proANP (*p* = 0.030) were higher in patients with SS-ILD than in patients with COPD. The LV cavity size was higher in the group of patients with COPD (*p* = 0.039). In our study, no statistically significant differences were found in the values of estimated systolic pressure in the pulmonary artery (eSPPA) in patients with COPD and SS-ILD; however, in patients with SS-ILD, this indicator was 1.6 times higher. The analysis of pCO_2_ and pO_2_, according to the gas composition of arterial blood, showed no statistically significant differences among patients with COPD and SS-ILD with HFpEF. However, the first quartiles of pO_2_ were low, and the third quartiles of pCO_2_ were higher than normal in both groups. Thus, it can be said that at least 25% of patients had hypoxia and/or hypercapnia, which reflects impaired alveolar ventilation.

## 4. Discussion

The causes of HFpEF development in patients with COPD are heterogeneous, making it extremely difficult to identify a single prevailing factor [12]. This complexity arises from the fact that several factors may simultaneously contribute to the development of COPD, and these are further aggravated by the onset of HFpEF. These factors alter the architecture of the bronchial tree, the lungs, and the structure of the myocardium [7,13]. Moreover, these complex interactions result in different clinical and structural phenotypes, supporting the need for a personalized medical approach that takes into account individual variability in disease mechanisms. In our study, we observed statistically significant patency disorders across all bronchial levels in patients with COPD and HFpEF compared to those without HFpEF. This indicates the development of generalized bronchial obstruction. Additionally, the enlargement of the LV cavity is likely associated with alveolar vasoconstriction and increased vascular stiffness [14,15,16,17]. Snigdha Jain et al. conducted an original study on the effect of COPD on the development of HFpEF, which found that patients with COPD and HFpEF exhibited increased aortic wall stiffness, myocardial fibrosis, and concentric LV hypertrophy [14]. This systemic remodeling of the cardiovascular system is likely driven by systemic inflammation, hypoxia in patients with COPD, and the side effects of m-anticholinergics, as well as the non-prescription of beta-blockers due to concerns about increasing bronchospasm [18,19,20]. These ideas underscore the importance of individualized pharmacological planning and risk–benefit assessments for patients with concurrent pulmonary and cardiac dysfunction.

In our study, patients with COPD experienced frequent exacerbations leading to hospitalizations (2–3 times a year or more). From this perspective, we believe that our patients experienced both systemic and local inflammation. Interestingly, despite the presence of HFpEF, there was no statistically significant difference in life quality scores between patients with and without HFpEF, according to the World Health Organization Quality of Life questionnaire. This suggests that in patients with chronic lung disease, the dominant burden on perceived quality of life may stem more from pulmonary symptoms than from cardiovascular dysfunction. From this perspective, we believe that our patients experienced both systemic and local inflammation. Chronic inflammation triggers oxidative stress and an increase in reactive oxygen species, which diminishes endothelial nitric oxide synthase (eNOS) activity and nitric oxide (NO) levels [21,22]. An increase in serum endothelin-1 concentrations further reduces NO bioavailability [23]. Existing bronchial obstruction in COPD, coupled with increased end-expiratory pressure, air trapping, and decreased lung tissue elasticity due to emphysema, results in increased chest resistance and intrathoracic pressure. This contributes to the development of respiratory failure and subsequent hypoxic vasoconstriction [24,25,26]. These biomarkers could serve as early indicators of subclinical cardiac damage in COPD, supporting the potential for biomarker-driven risk stratification and personalized monitoring strategies. Hypoxic pulmonary vasoconstriction exacerbates endothelial dysfunction through an imbalance of vasodilatory and vasoconstrictor regulatory mechanisms [27,28]. Locally developed endothelial dysfunction in the pulmonary vessels in COPD becomes systemic, affecting the microcirculatory networks, the coronary bed, and beyond [27]. Chronic persistent inflammation, oxidative stress, and endothelial dysfunction disrupt the balance between elastin and collagen (with elastin degradation and collagen accumulation), leading to increased arterial rigidity [29]. This limits the ability of blood vessels to expand and contract in response to pressure changes. Moreover, systemic inflammation, through the activation of proinflammatory cytokines, directly damages cardiomyocytes, stimulates cardiomyocyte and fibroblast growth and leads to myocardial hypertrophy, extracellular matrix disruption, and ultimately the concentric remodeling of the cardiac chambers [30,31,32]. In support of this, our study revealed elevated levels of cardiac biomarkers such as MR-proANP and high-sensitivity troponin in patients with HFpEF, indicating myocardial stress and subtle injury, even in the absence of overt cardiac symptoms. These findings align with the concept of low-grade, chronic myocardial involvement. These biomarkers could serve as early indicators of subclinical cardiac damage in COPD, supporting the potential for biomarker-driven risk stratification and personalized monitoring strategies.

Additionally, endothelial dysfunction can contribute to heart pressure overload, which, in combination with increased arterial wall stiffness, also leads to concentric remodeling [33]. The anatomical narrowing of the pulmonary veins due to vasoconstriction, combined with increased intrathoracic pressure, interferes with venous return and reduces the left ventricular preload and pericardial restriction, which can further affect LV function [34]. As LV contraction increases pressure in the pulmonary vessels due to hypoxic vasoconstriction, the LV must exert additional effort. If this process is prolonged, it leads to the compensatory hypertrophy of cardiomyocytes. Thus, we hypothesize that, among all the multifactorial conditions, hypoxia resulting from airflow limitation and pulmonary hyperinflation acts as the trigger for HFpEF development in patients with COPD.

Patients with SS-ILD are characterized by systemic vasculopathy, which involves vascular remodeling and angiogenesis in small pulmonary arteries [35]. This process may be driven by autoimmunity, leading to vessel wall inflammation and fibrosis, as well as endothelial dysfunction. Endothelial cells can become dysfunctional due to damage from autoantibodies, inflammatory mediators, oxidative stress, and increased endothelin-1 synthesis, which triggers vasoconstriction, increased vascular resistance, and endothelial-mesenchymal transition with uncontrolled collagen synthesis [36]. These mechanisms ultimately result in vascular stiffness, increased extracellular matrix deposition, and fibrosis formation [37]. Impaired gas exchange due to endothelial capillary membrane failure triggers chronic hypoxia [38], which can maintain and exacerbate existing pulmonary vasoconstriction, eventually leading to an irreversible increase in pulmonary arterial pressure [39]. In our study, we noted that eSPPA was significantly higher in the SS-ILD group. The development of pulmonary arterial hypertension (PAH) leads to an increase in right ventricular (RV) afterload [40]. At the early stages of PAH, the RA compensates for the increased isovolumetric pressure in the RV by increasing contractility and extensibility. However, as PAH progresses, the RV afterload intensifies, leading to RV dysfunction, which is difficult to assess through echocardiography. Consequently, we can only indirectly assess RV dysfunction by observing changes in RA size [41]. This highlights the importance of using advanced imaging techniques and biomarkers to better understand right heart disease and develop personalized treatment strategies for patients with SS-ILD and PAH. Previous studies of systemic sclerosis patients with diastolic dysfunction have found changes in RA size, without corresponding changes in the RV [42,43].

Simultaneously, inflammation and endothelial dysfunction occur in SS-ILD. The endothelial–mesenchymal transition is also initiated in the myocardial vascular bed. The progression of PAH likely exacerbates this process, ultimately contributing to the development of not only RV diastolic dysfunction but also LV dysfunction, which leads to the formation of HFpEF [44]. These mechanisms result in non-hypertrophic LV remodeling in SS-ILD. It is likely that hypoxic vasoconstriction increases pressure in the pulmonary circulation (in the pulmonary arteries), leading to increased RV afterload. The LV receives blood from the lungs through the pulmonary veins with normal or even reduced pressure and therefore does not experience additional load compared to the RV. Thus, in patients with SS-ILD, the right side of the heart faces an increased afterload due to PAH, while the left myocardium experiences dysfunction and remodeling due to systemic endothelial dysfunction and fibrosis, without hypertrophy.

The results of our study should be interpreted with the potential limitations in mind, which are based on the inclusion and exclusion criteria. This study did not include patients with mild or very severe COPD or those experiencing exacerbations. Additionally, the cohort was predominantly male in the COPD group, while it was mostly female in the SS-ILD group. Therefore, we cannot extrapolate the results to the entire population of COPD and SS-ILD patients, especially those with chronic lung disease (CLD). Moreover, patients with COPD exhibited more pronounced lung hyperinflation, which significantly complicated the echocardiographic assessment.

Nevertheless, by grouping the results according to pathogenetic trends, we identified two different mechanisms of HFpEF development in COPD, a hypoxic phenotype of HFpEF, and in SS-ILD, myocardial remodeling against the background of endothelial dysfunction and systemic fibrosis in the pulmonary artery system. Recognizing these phenotypes allows clinicians to focus on the early identification and management of HFpEF in patients with COPD and ILD. Recognizing these phenotypes forms the basis for phenotype-specific management strategies in clinical practice, enabling early diagnosis, individualized risk stratification, and personalized therapeutic interventions for patients with chronic lung diseases and HFpEF.

## 5. Conclusions

Our results suggest the existence of two different mechanisms in the development of HFpEF among patients with COPD and SS-ILD, underscoring the importance of personalized medicine in managing heart failure associated with chronic lung diseases. In patients with COPD, hypoxia—primarily due to airflow limitation and pulmonary hyperinflation—appeared to be the dominant contributor to HFpEF development. Conversely, in SS-ILD patients, myocardial dysfunction and structural remodeling were linked to endothelial dysfunction and systemic fibrosis within the pulmonary vasculature. By utilizing targeted diagnostic criteria to identify these subgroups, we can enable the earlier detection of HFpEF and develop individualized treatment plans, ultimately improving patient outcomes and optimizing personalized healthcare resource utilization in this diverse population.

## Figures and Tables

**Table 1:** Characteristics of patients with CLD with/without HFpEF.

Indicator	Without HFpEF	With HFpEF	*p*
Age, years	54 (48; 59)	58 (51.5; 62.5)	0.041
Weight, kg	65 (56; 80)	65 (60; 71.5)	0.908
Height, cm	167 (160; 172)	167.5 (161; 174)	0.956
BMI, kg/m^2^	23.505 (20.72; 28.7)	24.26 (21.415; 28.25)	0.631
RR up to 6MWT	19 (18; 20)	19 (18; 21.5)	0.600
RR after 6MWT	23 (21; 26)	23.5 (21; 26)	0.768
Distance 6MWT, meters	265 (200; 340)	265 (182.5; 330)	0.464
SaO_2_ up to 6MWT	96 (95; 97)	95.5 (94; 97.5)	0.697
SaO_2_ after 6MWT	94 (89; 96)	94 (89; 96)	0.647
HR up to 6MWT	80 (73; 86)	77 (73; 83)	0.347
HR after 6MWT	91 (85; 100)	90.5 (88; 98)	0.945
SBP up to 6MWT	120 (110; 120)	120 (110; 130)	0.343
SBP after 6MWT	120 (120; 130)	130 (120; 140)	0.067
DBP up to 6MWT	70 (70; 80)	75 (70; 80)	0.911
DBP after 6MWT	80 (80; 90)	80 (70; 90)	0.816
Borg scale up to 6MWT, points	1 (0.5; 2)	2 (1; 2.5)	0.075
Borg scale after 6MWT, points	3 (2; 4)	3 (3; 5)	0.359
WHOQOL:1, points	21 (19; 24)	21 (18; 22.5)	0.397
WHOQOL:2, points	20 (17; 22)	20.5 (17.5; 22.5)	0.625
WHOQOL:3, points	11 (9; 12)	11 (8.5; 12)	0.706
WHOQOL:4, points	26 (23; 31)	29.5 (25.5; 34)	0.117
FVC, %	71.75 (54.6; 95.1)	64.3 (46.5; 76.3)	0.104
FEV1, %	61.7 (41.2; 84)	49.45 (31.45; 82.9)	0.178
FVC/FEV1, %	75.8 (54.3; 87)	73.45 (48.85; 89.7)	0.801
MEF 25, %	49.55 (21; 92.1)	38.75 (12.75; 76.05)	0.325
MEF 50, %	46.05 (18.7; 77)	31.2 (13.25; 73.15)	0.488
MEF 75, %	38.1 (21.9; 65.7)	28.85 (17.6; 60.75)	0.440
Galectin-3, ng/mL	17.1 (14; 21.2)	19.65 (15.8; 25.65)	0.177
Endothelin-1, pg/mL	33.637 (17.7869; 50.026)	35.0815 (15.8977; 58.274)	0.864
MR-proANP, pmol/L	22.4324 (9.1228; 40.6368)	52.4565 (22.0088; 93.6059)	0.010
hsTnT, pg/mL	1.0706 (0.632; 1.7866)	1.6383 (1.0825; 3.0588)	0.015
LVEF	63 (58; 67)	64.5 (58; 67.5)	0.694
LV cavity size, mm	39 (35; 43.5)	40 (36; 45)	0.324
Longitudinal dimension of the LA, mm	46 (39; 52)	49 (44; 59)	0.112
LA area, mm	15 (12.2; 18.9)	17.1 (12.8; 19.8)	0.473
Volume of LA, mm	32.5 (25; 48)	48 (30; 59.5)	0.128
Size of the RV, mm	29 (25; 33)	26 (23; 30)	0.145
Longitudinal size of RA, mm	41 (38; 46)	45.5 (43; 49)	0.027
RA area, mm	12.1 (9.6; 17.8)	13.7 (11.3; 15)	0.478
Volume of RA, mm	23 (16; 32)	36 (26; 38)	0.058
EDV, mL	79 (64; 91)	79 (66; 90)	0.784
ESV, mL	28 (21; 33)	28 (24; 34)	0.777
SV, mL	49 (40.5; 56)	50 (38; 56)	0.768
Pulmonary artery, mm	22 (21; 24)	24 (21.5; 25.5)	0.321
eSPPA, mmHg	20 (17; 28.75)	33.5 (23; 53.75)	0.063
Capnography SaO_2_, %	95 (92; 96)	95.5 (93.5; 96.75)	0.496
Capnography of heart rate	84 (78; 96.75)	76 (73; 83.5)	0.337
Capnography of respiratory rate	21 (18; 22)	24 (17.25; 27)	0.868
Capnography CO_2_, mmHg	30 (26.25; 35)	25 (17.25; 35)	0.229
pCO_2_, mmHg	39 (34.25; 44.43)	36.8 (31.73; 45.1)	0.903
pO_2_, mmHg	85 (70.23; 103.5)	43.45 (29.6; 80.87)	0.755

BMI—body mass index; RR—respiratory rate; HR—heart rate; 6MWT—6 min walk test; SBP—systolic blood pressure; DBP—diastolic blood pressure; WHOQOL:1—physical health dimension of the World Health Organization Quality of Life questionnaire; WHOQOL:2—psychological health dimension of the World Health Organization Quality of Life questionnaire; WHOQOL:3 social relationship dimension of the World Health Organization Quality of Life questionnaire; WHOQOL:4—environment dimension of the World Health Organization Quality of Life questionnaire; FVC—forced vital capacity; FEV1—forced expiratory volume in 1 s; MEF—maximum expiratory flow rate (MEF); LV—left ventricle; LA—left atrium; RA—right atrium; RV—right ventricle; EDV—end-diastolic volume; ESV—end-systolic volume; SV—stroke volume; eSPPA—estimated systolic pressure in the pulmonary artery; TAPSE—tricuspid annular plane systolic excursion; pCO_2_—partial pressure of carbon dioxide; pO_2_—partial pressure of oxygen, 6 min walk test; SBP—systolic blood pressure; DBP—diastolic blood pressure,; CAT—COPD Assessment Test; FVC—forced vital capacity; FEV1—forced expiratory volume in 1 s; MEF—maximum expiratory flow rate (MEF); LV—left ventricle; LA—left atrium; RA—right atrium; RV—right ventricle; EDV—end diastolic volume; ESV—end systolic volume; SV—stroke volume; eSPPA—estimated systolic pressure in the pulmonary artery; TAPSE—tricuspid annular plane systolic excursion; pCO_2_—partial pressure of carbon dioxide; pO_2_—partial pressure of oxygen.

**Table 2:** Characteristics of patients with COPD with/without HFpEF.

Indicator	Without HFpEF	With HFpEF	*p*
Volume of LA, mm	32 (25; 46)	59 (52; 69.5)	0.020

LA—left atrium.

**Table 3:** Characteristics of patients with SS-ILD with/without HFpEF.

Indicator	Without HFpEF	With HFpEF	*p*
SBP up to 6MWT	110 (110; 120)	120 (110; 130)	0.040
SBP after 6MWT	120 (120; 130)	140 (120; 145)	0.006
Borg scale up to 6MWT, points	1 (0; 2)	2 (1; 3)	0.008
MR-proANP, pmol/L	26.68 (14.64; 59.13)	92.47 (37.67; 144.24)	0.006
Longitudinal size of RA, mm	41 (36; 43)	46 (43; 47)	0.005

6MWT—6 min walk test; SBP—systolic blood pressure.

**Table 4:** Characteristics of patients with HFpEF in COPD and SS-ILD.

Indicator	Without HFpEF	With HFpEF	*p*
Age, years	59 (57; 63)	57 (48; 62)	0.361
Weight, kg	67 (65; 80)	60 (56; 71)	0.033
Height, cm	170 (168; 173)	164 (156; 175)	0.209
BMI, kg/m^2^	24.9 (21.45; 28.1)	23.62 (19.92; 28.4)	0.732
Respiratory rate up to 6MWT	21 (19; 23)	19 (18; 20)	0.034
Respiratory rate after 6MWT	26 (22; 26)	22 (20; 25)	0.107
Distance 6MWT, meters	190 (101; 300)	320 (220; 350)	0.062
SaO_2_ up to 6MWT	96.5 (95; 98)	96.5 (94.5; 98)	0.957
SaO_2_ after 6MWT	95.5 (93; 97)	95.5 (91; 96)	0.706
HR up to 6MWT	80 (74; 87)	74 (72; 80)	0.359
HR after 6MWT	90 (80; 98)	92 (88; 98)	0.542
SBP up to 6MWT	110 (101; 120)	120 (110; 130)	0.121
SBP after 6MWT	130 (101; 130)	140 (120; 145)	0.044
DBP up to 6MWT	80 (70; 80)	70 (70; 80)	0.635
DBP after 6MWT	80 (70; 90)	80 (70; 90)	0.509
Borg scale up to 6MWT, points	2 (1; 2)	2 (1; 3)	0.368
Borg scale after 6MWT, points	4 (3; 5)	3 (3; 6)	0.697
WHOQOL:1, points	19 (17; 21)	22 (18; 23)	0.223
WHOQOL:2, points	11 (7; 12)	12 (9; 14)	0.282
WHOQOL:3, points	28 (26; 31)	32 (25; 34)	0.338
WHOQOL:4, points	65 (48; 71.9)	62.5 (43; 92)	0.909
FVC, %	35.8 (24; 49.6)	66.9 (38.6; 86.7)	0.044
FEV1, %	50.2 (44.3; 62.2)	81 (73.9; 98)	0.007
FVC/FEV1, %	13.5 (9; 26)	75.1 (41.8; 112)	0.003
MEF 25, %	14 (10.3; 15.8)	73 (39.5; 118)	0.004
MEF 50, %	18.1 (15.2; 20.4)	49 (31.1; 117.7)	0.007
MEF 75, %	16 (15.6; 19.5)	22.4 (18.7; 30.8)	0.068
Galectin-3, ng/mL	264 (136.7; 269.4)	222 (177.2; 552.4)	0.676
Endothelin-1, pg/mL	17.6177 (15.08; 36.476)	41.969 (31.3192; 76.59)	0.030
MR-proANP, pmol/L	23.2632 (12.5439; 66.3221)	92.4742 (37.6731; 144.2445)	0.030
hsTnT, pg/mL	1.5062 (1.2235; 2.7529)	1.7704 (0.9414; 3.3647)	0.879
LV cavity size, mm	45 (41; 46)	37 (36; 41)	0.039
Longitudinal dimension of the LA, mm	52.5 (37; 56)	48 (45; 59)	0.953
LA area, mm	18.25 (17.95; 20.35)	14.45 (11.9; 18.7)	0.126
Volume of LA, mm	59 (52; 69.5)	35.5 (24.5; 53.5)	0.051
Size of the RV, mm	28 (24; 31)	26 (23; 30)	0.556
Longitudinal size of RA, mm	44 (44; 49)	46 (43; 47)	0.739
RA area, mm	13.7 (13.1; 15)	12.75 (11.2; 15.5)	0.796
Volume of RA, mm	36 (27; 45)	31.5 (22; 38)	0.519
EDV, mL	84 (73; 92)	78 (66; 88)	0.480
ESV, mL	32 (28; 38)	25 (24; 29)	0.195
SV, mL	51 (45; 56)	50 (38; 54)	0.906
Pulmonary artery, mm	24.5 (23; 25)	23.5 (20.5; 26.5)	0.799
eSPPA, mmHg	27.5 (20; 35)	46 (32; 60)	0.567
Capnography CO_2_, mmHg	25 (18; 32)	26.5 (17; 36)	0.176
pCO_2_, mmHg	39.4 (38.9; 53)	34.2 (31.68; 47)	0.465
pO_2_, mmHg	90.83 (48; 95.5)	87 (51; 93.3)	0.855

BMI—body mass index; RR—respiratory rate; HR—heart rate; 6MWT—6 min walk test; SBP—systolic blood pressure; DBP—diastolic blood pressure; WHOQOL:1—physical health dimension of the World Health Organization Quality of Life questionnaire; WHOQOL:2—psychological health dimension of the World Health Organization Quality of Life questionnaire; WHOQOL:3 social relationship dimension of the World Health Organization Quality of Life questionnaire; WHOQOL:4—environment dimension of the World Health Organization Quality of Life questionnaire; FVC—forced vital capacity; FEV1—forced expiratory volume in 1 s; MEF—maximum expiratory flow rate (MEF); LV—left ventricle; LA—left atrium; RA—right atrium; RV—right ventricle; EDV—end-diastolic volume; ESV—end-systolic volume; SV—stroke volume; eSPPA—estimated systolic pressure in the pulmonary artery; TAPSE—tricuspid annular plane systolic excursion; pCO_2_—partial pressure of carbon dioxide; pO_2_—partial pressure of oxygen, 6 min walk test; SBP—systolic blood pressure; DBP—diastolic blood pressure; CAT—COPD Assessment Test; FVC—forced vital capacity; FEV1—forced expiratory volume in 1 s; MEF—maximum expiratory flow rate (MEF); LV—left ventricle; LA—left atrium; RA—right atrium; RV—right ventricle; EDV—end diastolic volume; ESV—end systolic volume; SV—stroke volume; eSPPA—estimated systolic pressure in the pulmonary artery; TAPSE—tricuspid annular plane systolic excursion; pCO_2_—partial pressure of carbon dioxide; pO_2_—partial pressure of oxygen.

## Data Availability

The Dataset is available upon request from the authors.

## Supplementary Materials

The following supporting information can be downloaded at https://www.mdpi.com/article/10.3390/jpm15050206/s1.

## Abbreviations

The following abbreviations are used in this manuscript:

CLD	Chronic lung disease;
CHF	Chronic heart failure;
HFrEF	HF with reduced ejection fraction;
HFpEF	HF with preserved ejection fraction;
6MWT	6 min walk test;
SBP	Systolic blood pressure;
DBP	Diastolic blood pressure;
CAT	COPD Assessment Test;
WHOQOL:1	Physical health dimension of the World Health Organization Quality of Life questionnaire;
WHOQOL:2	Psychological health dimension of the World Health Organization Quality of Life questionnaire;
WHOQOL:3	Social relationships dimension of the World Health Organization Quality of Life questionnaire;
WHOQOL:4	Environment dimension of the World Health Organization Quality of Life questionnaire;
HR	Heart rate;
RR	Respiratory rate;
FVC	Forced vital capacity;
FEV1	Forced expiratory volume in 1 s;
MEF	Maximum expiratory flow rate;
LV	Left ventricle;
LA	Left atrium;
RA	Right atrium;
RV	Right ventricle;
EDV	End diastolic volume;
ESV	End systolic volume;
SV	Stroke volume;
eSPPA	Estimated systolic pressure in the pulmonary artery;
TAPSE	Tricuspid annular plane systolic excursion;
BODE-index	B—body mass index; O—obstruction (obstruction); D—dyspnea (shortness of breath); E—exercise tolerance (tolerance to physical activity);
ADO index	Age, dyspnea, and airflow obstruction;
pCO_2_	Partial pressure of carbon dioxide;
pO_2_	Partial pressure of oxygen.

## References

[1] Lin C.H., Yeh J.K., Lin T.Y., Lo Y.L., Chang B.J., Ju J.S., Chiu T.H., Tung P.H., Huang Y.J., Lin S.M. (2023). Influence of chronic obstructive pulmonary disease on long-term hospitalization and mortality in patients with heart failure with reduced ejection fraction. BMC Pulm. Med..

[2] Ehteshami-Afshar S., Mooney L., Dewan P., Desai A.S., Lang N.N., Lefkowitz M.P., Petrie M.C., Rizkala A.R., Rouleau J.L., Solomon S.D. (2021). Clinical characteristics and outcomes of patients with heart failure with reduced ejection fraction and chronic obstructive pulmonary disease: Insights from PARADIGM-HF. J. Am. Heart Assoc..

[3] Zhu A., Hu M., Ge D., Zhang X., Zhang J., Wang Y., Yao X., Liu J. (2025). Prevalence and clinical correlates of chronic obstructive pulmonary disease in heart failure patients: A cross-sectional study in China. Front. Med..

[4] Hesse K., Bourke S., Steer J. (2022). Heart failure in patients with COPD exacerbations: Looking below the tip of the iceberg. Respir. Med..

[5] Becher P.M., Lindberg F., Benson L., Hage C., Dahlström U., Rosenkranz S., Cosentino F., Rosano G.M., Blankenberg S., Kirchhof P. (2024). Phenotyping patients with chronic obstructive pulmonary disease and heart failure. ESC Heart Fail..

[6] Plachi F., Balzan F.M., Sanseverino R.A., Palombini D.V., Marques R.D., Clausell N.O., Knorst M.M., Neder J.A., Berton D.C. (2018). Characteristics associated with mortality in patients with chronic obstructive pulmonary disease (COPD)-heart failure coexistence. Prim. Health Care Res. Dev..

[7] Mooney L., Hawkins N.M., Jhund P.S., Redfield M.M., Vaduganathan M., Desai A.S., Rouleau J.L., Minamisawa M., Shah A.M., Lefkowitz M.P. (2021). Impact of Chronic Obstructive Pulmonary Disease in Patients With Heart Failure With Preserved Ejection Fraction: Insights From PARAGON-HF. J. Am. Heart Assoc..

[8] Khalid K., Padda J., Komissarov A., Colaco L.B., Padda S., Khan A.S., Campos V.M., Jean-Charles G. (2021). The Coexistence of Chronic Obstructive Pulmonary Disease and Heart Failure. Cureus.

[9] Oliveira F.M., Rei A.L., Oliveira M.I., Almeida I., Santos M. (2022). Prevalence and prognostic significance of heart failure with preserved ejection fraction in systemic sclerosis. Future Cardiol..

[10] Fell B.L., Hanekom S., Heine M. (2021). Six-minute walk test protocol variations in low-resource settings—A scoping review. S. Afr. J. Physiother..

[11] Egashira K., Sueta D., Komorita T., Yamamoto E., Usuku H., Tokitsu T., Fujisue K., Nishihara T., Oike F., Takae M. (2022). HFA-PEFF scores: Prognostic value in heart failure with preserved left ventricular ejection fraction. Korean J. Intern. Med..

[12] Khan S.S., Kalhan R. (2022). Comorbid Chronic Obstructive Pulmonary Disease and Heart Failure: Shared Risk Factors and Opportunities to Improve Outcomes. Ann. Am. Thorac. Soc..

[13] Xu S., Gu Z., Zhu W., Feng S. (2024). Association of COPD with adverse outcomes in heart failure patients with preserved ejection fraction. ESC Heart Fail..

[14] Jain S., Obeid M.J., Yenigalla S., Paravathaneni M., Gadela N.V., Singh G., Kulkarni V., Kondaveety S., Gade K.C., Lee J. (2021). Impact of chronic obstructive pulmonary disease in heart failure with preserved ejection fraction. Am. J. Cardiol..

[15] Borlaug B.A., Kass D.A. (2008). Ventricular–vascular interaction in heart failure. Heart Fail. Clin..

[16] Myronenko O., Foris V., Crnkovic S., Olschewski A., Rocha S., Nicolls M.R., Olschewski H. (2023). Endotyping COPD: Hypoxia-inducible factor-2 as a molecular “switch” between the vascular and airway phenotypes?. Eur. Respir. Rev..

[17] Abdo W.F., Heunks L.M. (2012). Oxygen-induced hypercapnia in COPD: Myths and facts. Crit. Care.

[18] Pugliese N.R., Pellicori P., Filidei F., De Biase N., Maffia P., Guzik T.J., Masi S., Taddei S., Cleland J.G.F. (2022). Inflammatory pathways in heart failure with preserved left ventricular ejection fraction: Implications for future interventions. Cardiovasc. Res..

[19] Peh Z.H., Dihoum A., Hutton D., Arthur J.S.C., Rena G., Khan F., Lang C.C., Mordi I.R. (2023). Inflammation as a therapeutic target in heart failure with preserved ejection fraction. Front. Cardiovasc. Med..

[20] Nakamura K., Akagi S., Ejiri K., Taya S., Saito Y., Kuroda K., Takaya Y., Toh N., Nakayama R., Katanosaka Y. (2025). Pathophysiology of Group 3 Pulmonary Hypertension Associated with Lung Diseases and/or Hypoxia. Int. J. Mol. Sci..

[21] Barnes P.J. (2022). Oxidative Stress in Chronic Obstructive Pulmonary Disease. Antioxidants.

[22] McGuinness A.J.A., Sapey E. (2017). Oxidative stress in COPD: Sources, markers, and potential mechanisms. J. Clin. Med..

[23] Theodorakopoulou M.P., Alexandrou M.E., Bakaloudi D.R., Pitsiou G., Stanopoulos I., Kontakiotis T., Boutou A.K. (2021). Endothelial dysfunction in COPD: A systematic review and meta-analysis of studies using different functional assessment methods. ERJ Open Res..

[24] Higham A., Quinn A.M., Cançado J.E.D., Singh D. (2019). The pathology of small airways disease in COPD: Historical aspects and future directions. Respir. Res..

[25] Papandrinopoulou D., Tzouda V., Tsoukalas G. (2012). Lung compliance and chronic obstructive pulmonary disease. Pulm. Med..

[26] O’donnell D.E., Webb K.A., Neder J.A. (2015). Lung hyperinflation in COPD: Applying physiology to clinical practice. COPD Res. Pract..

[27] Screm G., Mondini L., Salton F., Confalonieri P., Trotta L., Barbieri M., Romallo A., Galantino A., Hughes M., Lerda S. (2024). Vascular Endothelial Damage in COPD: Where Are We Now, Where Will We Go?. Diagnostics.

[28] Siragusa S., Natali G., Nogara A.M., Trevisani M., Lagrasta C.A.M., Pontis S. (2023). The role of pulmonary vascular endothelium in chronic obstructive pulmonary disease (COPD): Does endothelium play a role in the onset and progression of COPD?. Explor. Med..

[29] Weir-McCall J.R., Struthers A.D., Lipworth B.J., Houston J.G. (2015). The role of pulmonary arterial stiffness in COPD. Respir. Med..

[30] Rabe K.F., Hurst J.R., Suissa S. (2018). Cardiovascular disease and COPD: Dangerous liaisons?. Eur. Respir. Rev..

[31] Pelà G., Calzi M.L., Pinelli S., Andreoli R., Sverzellati N., Bertorelli G., Goldoni M., Chetta A.A. (2016). Left ventricular structure and remodeling in patients with COPD. Int. J. Chronic Obstr. Pulm. Dis..

[32] Leonardi N.T., Tomaz C.d.S.R., Kabbach E.Z., Heubel A.D., Schafauser N.S., Kawakami D.M.d.O., Borghi-Silva A., Roscani M.G., Castello-Simões V., Mendes R.G. (2024). Left ventricular concentric remodeling in COPD patients: A cross-sectional observational study. Med. Clin. (Engl. Ed.).

[33] Zeder K., Marsh L.M., Avian A., Brcic L., Birnhuber A., Douschan P., Foris V., Sassmann T., Hoetzenecker K., Boehm P.M. (2024). Compartment-specific remodeling patterns in end-stage chronic obstructive pulmonary disease with and without severe pulmonary hypertension. J. Heart Lung Transplant..

[34] Alvarado A.C., Pinsky M.R. (2023). Cardiopulmonary interactions in left heart failure. Front. Physiol..

[35] Ackermann M., Werlein C., Plucinski E., Leypold S., Kühnel M.P., Verleden S.E., Khalil H.A., Länger F., Welte T., Mentzer S.J. (2024). The role of vasculature and angiogenesis in respiratory diseases. Angiogenesis.

[36] Pulito-Cueto V., Genre F., López-Mejías R., Mora-Cuesta V.M., Iturbe-Fernández D., Portilla V., Mora-Gil M.S., Ocejo-Vinyals J.G., Gualillo O., Blanco R. (2023). Endothelin-1 as a Biomarker of Idiopathic Pulmonary Fibrosis and Interstitial Lung Disease Associated with Autoimmune Diseases. Int. J. Mol. Sci..

[37] Low A., George S., Howard L., Bell N., Millar A., Tulloh R.M.R. (2018). Lung Function, Inflammation, and Endothelin-1 in Congenital Heart Disease–Associated Pulmonary Arterial Hypertension. J. Am. Heart Assoc..

[38] Bian F., Lan Y.-W., Zhao S., Deng Z., Shukla S., Acharya A., Donovan J., Le T., Milewski D., Bacchetta M. (2023). Lung endothelial cells regulate pulmonary fibrosis through FOXF1/R-Ras signaling. Nat. Commun..

[39] Sakao S., Tanabe N., Tatsumi K. (2019). Hypoxic Pulmonary Vasoconstriction and the Diffusing Capacity in Pulmonary Hypertension Secondary to Idiopathic Pulmonary Fibrosis. J. Am. Heart Assoc..

[40] Ruffenach G., Hong J., Vaillancourt M., Medzikovic L., Eghbali M. (2020). Pulmonary hypertension secondary to pulmonary fibrosis: Clinical data, histopathology and molecular insights. Respir. Res..

[41] Bech-Hanssen O., Astengo M., Fredholm M., Bergh N., Hjalmarsson C., Polte C.L., Ricksten S., Bollano E. (2021). Grading right ventricular dysfunction in left ventricular disease using echocardiography: A proof of concept using a novel multiparameter strategy. ESC Heart Fail..

[42] D’andrea A., D’alto M., Di Maio M., Vettori S., Benjamin N., Cocchia R., Argiento P., Romeo E., Di Marco G., Russo M.G. (2016). Right atrial morphology and function in patients with systemic sclerosis compared to healthy controls: A two-dimensional strain study. Clin. Rheumatol..

[43] Vos J.L., Butcher S.C., Fortuni F., Galloo X., Rodwell L., Vonk M.C., Bax J.J., van Leuven S.I., de Vries-Bouwstra J.K., Snoeren M. (2022). The prognostic value of right atrial and right ventricular functional parameters in systemic sclerosis. Front. Cardiovasc. Med..

[44] Singh A., Bhatt K.S., Nguyen H.C., Frisbee J.C., Singh K.K. (2024). Endothelial-to-mesenchymal transition in cardiovascular pathophysiology. Int. J. Mol. Sci..

