# Supplementary material for: Features of Heart Failure with Preserved Ejection Fraction in Patients with Chronic Obstructive Pulmonary Disease and Systemic Sclerosis-Associated Interstitial Lung Diseases
Source: J Pers Med. 2025 May 20;15(5):206. doi: 10.3390/jpm15050206 (PMC12112798; doi:10.3390/jpm15050206)
Supplement: Supplementary file 1 [file jpm-15-00206-s001.zip › jpm-3600321-supplementary.pdf]

Table S1. Characteristics of patients with COPD with/without HFpEF

Indicator	Without HFpEF	With HFpEF	p
Age, years	56 (49;61)	59 (57;63)	0.157
Weight, kg	65 (57;80)	67 (65;80)	0.456
Height, cm	172 (167;175)	170 (168;173)	0.533
BMI, kg/m^2	22.59 (19.59;28.07)	24.9 (21.45;28.1)	0.367
Respiratory rate up to 6MWT	20 (19;21)	21 (19;23)	0.193
Respiratory rate after 6MWT	24 (22;26)	26 (22;26)	0.525
Distance 6MWT, meters	240 (160;320)	190 (102;300)	0.250
SaO2 up to 6MWT	96 (94;96)	95 (93;97)	0.980
SaO2 after 6MWT	89 (88;95)	93 (89;96)	0.369
HR up to 6MWT	82 (77;88)	80 (74;87)	0.486
HR after 6MWT	95 (85;104)	90 (80;98)	0.286
SBP up to 6MWT	120 (110;120)	110 (102;120)	0.414
SBP after 6MWT	125 (120;130)	130 (102;130)	0.685
DBP up to 6MWT	80 (70;80)	80 (70;80)	0.923
DBP after 6MWT	80 (80;90)	80 (70;90)	0.536
Borg scale up to 6MWT, points	2 (1;2)	2 (1;2)	0.925
Borg scale after 6MWT, points	4 (3;5)	4 (3;5)	0.966
CAT, points	25 (14;32)	17 (15;29)	0.862
WHOQOL:1, points	21 (18;22)	19 (18;22)	0.496
WHOQOL:2, points	19 (16;21)	19 (17;21)	0.822
WHOQOL:3, points	10 (9;12)	11 (7;12)	0.789
WHOQOL:4, points	28 (26;31)	25 (23;27)	0.053
FVC, %	72.1 (54.6;97.4)	65 (48;71.9)	0.342
FEV1, %	45.1 (31.3;76.3)	35.8 (24;49.6)	0.167
FVC/FEV1, %	56.1 (38.8;72.7)	50.2 (44.3;62.2)	0.557
MEF 25, %	28.9 (12.6;48.6)	13.5 (9;26)	0.095
MEF 50, %	21.1 (11.4;41.7)	14 (10.3;15.8)	0.132
MEF 75, %	23 (17;38.1)	18.1 (15.2;20.4)	0.112
Galectin-3, ng / ml	15.9 (13.7;18.4)	16 (15.6;19.5)	0.869
Endothelin-1, pg / ml	28.2163 (17.6741;46.6394)	17.6177 (15.08;36.476)	0.098
MR-proANP, pmol /l	17.895 (5.7018;32.0429)	23.2632 (12.5439;66.3221)	0.342
hsTnT, pg / ml	1.0706 (0.6118;1.6824)	1.5062 (1.2235;2.7529)	0.069
LV cavity size, mm	41 (35;45)	45 (41;46)	0.150
Longitudinal dimension of the LA, mm	47 (39;51)	52.5 (37;56)	0.380
LA area, mm	16.3 (12.4;21.3)	18.25 (17.95;20.35)	0.239
Volume of LA, mm	32 (25;46)	59 (52;69.5)	0.020
Size of the RV, mm	31 (27.5;37)	28 (24;31)	0.147
Longitudinal size of RA, mm	43.5 (39;47)	44 (44;49)	0.665
RA area, mm	14.15 (10.4;27.35)	13.7 (13.1;15)	0.962
Volume of RA, mm	26 (19;32)	36 (27;45)	0.162
EDV, ml	82 (63;93)	84 (73;92)	0.772
ESV, ml	28 (22.5;35.5)	32 (28;38)	0.386
SV, ml	54 (36;58)	51 (45;56)	0.915

Pulmonary artery, mm	23 (22;25)	24.5 (23;25)	0.568
eSPPA, mmHg	22 (20;29)	27.5 (20;35)	0.199
Capnography CO_2, mmHg	25 (18;32)	39 (35.9;44.35)	0.114
BODE	6 (4;8)	6 (3;7)	0.591
ADO	2 (1;5)	5 (2;6)	0.172
pCO_2, mmHg	39 (35.95;44.35)	35.15 (30.9;39.4)	0.498
pO_2, mmHg	77 (69.95;104.1)	59.17 (27.5;90.83)	0.817

BMI - Body mass index, RR-Respiratory rate, HR -Heart rate, 6MWT - 6-minute walk test, SBP – systolic blood pressure, DBP – diastolic blood pressure, CAT - COPD Assessment Test, WHOQOL:1 - physical health of World Health Organization Quality of Life questionnaire, WHOQOL:2 - psychological health of World Health Organization Quality of Life questionnaire, WHOQOL:3 social relationships of World Health Organization Quality of Life questionnaire, WHOQOL:4 – environment of World Health Organization Quality of Life questionnaire, FVC – forced vital capacity, FEV1 – forced expiratory volume in 1 second, MEF – maximum expiratory flow rate (MEF), LV – left ventricle, LA – left atrium, RA – right atrium, RV – right ventricle, EDV – end diastolic volume, ESV – end systolic volume, SV – stroke volume, eSPPA – estimated systolic pressure in the pulmonary artery, TAPSE - Tricuspid annular plane systolic excursion, BODE-index-B – body mass index, O – obstruction (obstruction) D – dyspnea (shortness of breath), E – exercise tolerance (tolerance to physical activity), ADO index - age, dyspnoea, airflow obstruction, pCO_2 - partial pressure of carbon dioxide, pO_2 - partial pressure of oxygen

Table S2. Characteristics of patients with SS-ILD with/without HFpEF

**Indicator**	**Without HFpEF**	**With HFpEF**	**p**
Age, years	52 (43;57)	57 (48;62)	0.088
Weight, kg	65 (56;75)	60 (56;71)	0.460
Height, cm	162 (157;165)	164 (156;175)	0.416
BMI, kg/m^2	24 (21;29.36)	23.62 (19.92;28.4)	0.830
Respiratory rate up to 6MWT	19 (18;20)	19 (18;20)	0.970
Respiratory rate after 6MWT	21 (20;26)	22 (20;25)	0.876
Distance 6MWT, meters	295 (240;360)	320 (220;350)	0.865
SaO2 up to 6MWT	97 (95;98)	96 (94;98)	0.491
SaO2 after 6MWT	95 (94;97)	96 (89;96)	0.516
HR up to 6MWT	76 (71;84)	74 (72;80)	0.923
HR after 6MWT	89 (84;94)	92 (88;98)	0.105
SBP up to 6MWT	110 (110;120)	120 (110;130)	0.040
SBP after 6MWT	120 (120;130)	140 (120;145)	0.006
DBP up to 6MWT	70 (70;80)	70 (70;80)	0.813
DBP after 6MWT	80 (80;90)	80 (70;90)	0.781
Borg scale up to 6MWT, points	1 (0;2)	2 (1;3)	0.008
Borg scale after 6MWT, points	3 (1;4)	3 (3;6)	0.215
WHOQOL:1, points	20 (18;24)	22 (18;23)	0.876
WHOQOL:2, points	12 (10;14)	12 (9;14)	0.633
WHOQOL:3, points	29 (24;34)	32 (25;34)	0.784
WHOQOL:4, points	71.4 (54.9;90)	62.5 (43;92)	0.212

FVC, %	71.9 (60.1;85.6)	66.9 (38.6;86.7)	0.590
FEV1, %	82.6 (77;90.4)	81 (73.9;98)	0.994
FVC/FEV1, %	76.6 (50.5;101)	75.1 (41.8;112)	0.757
MEF 25, %	63.9 (50.6;84.7)	73 (39.5;118)	0.690
MEF 50, %	52.5 (38;80.3)	49 (31.1;117.7)	0.848
MEF 75, %	18.7 (14.3;22.4)	22.4 (18.7;30.8)	0.073
Galectin-3, ng / ml	54.5 (26.5;130.5)	222 (177.2;552.4)	0.000
Endothelin-1, pg / ml	37.7187 (19.153;50.9058)	41.969 (31.3192;76.59)	0.259
MR-proANP, pmol /l	26.68 (14.64;59.13)	92.47 (37.67;144.24)	0.006
hsTnT, pg / ml	1.071 (0.74;1.78)	1.77 (0.94;3.36)	0.114
LV cavity size, mm	37 (35;41)	37 (36;41)	0.665
Longitudinal dimension of the LA, mm	46 (40;54)	48 (45;59)	0.143
LA area, mm	14.1 (11.4;16.7)	14.45 (11.9;18.7)	0.720
Volume of LA, mm	34 (24;48)	35.5 (24.5;53.5)	0.815
Size of the RV, mm	26 (24;30.5)	26 (23;30)	0.601
Longitudinal size of RA, mm	41 (36;43)	46 (43;47)	0.005
RA area, mm	10.8 (9.3;13.2)	12.75 (11.2;15.5)	0.172
Volume of RA, mm	21.5 (16;32.5)	31.5 (22;38)	0.113
EDV, ml	72 (64.5;83.5)	78 (66;88)	0.896
ESV, ml	25 (21;32)	25 (24;29)	0.963
SV, ml	48 (41;55)	50 (38;54)	0.736
Pulmonary artery, mm	22 (20.5;23)	23.5 (20.5;26.5)	0.330
eSPPA, mmHg	20 (16;22)	37 (32;50)	0.194
Capnography CO_2, mmHg	32.5 (28;37)	34 (24.5;36.5)	0.831
pCO_2, mmHg	40 (33; 44.6)	34.2 (31.68;47)	0.918
pO_2, mmHg	86 (67;98)	87 (51;93.3)	0.776

BMI - Body mass index, RR-Respiratory rate, HR -Heart rate, 6MWT - 6-minute walk test, SBP – systolic blood pressure, DBP – diastolic blood pressure, WHOQOL:1 - physical health of World Health Organization Quality of Life questionnaire, WHOQOL:2 - psychological health of World Health Organization Quality of Life questionnaire, WHOQOL:3 social relationships of World Health Organization Quality of Life questionnaire, WHOQOL:4 – environment of World Health Organization Quality of Life questionnaire, FVC – forced vital capacity, FEV1 – forced expiratory volume in 1 second, MEF - maximum expiratory flow rate (MEF), LV – left ventricle, LA – left atrium, RA – right atrium, RV – right ventricle, EDV - end-diastolic volume, ESV - end-systolic volume, SV – stroke volume, eSPPA - estimated systolic pressure in the pulmonary artery, TAPSE - Tricuspid annular plane systolic excursion, pCO_2 - partial pressure of carbon dioxide, pO_2 - partial pressure of oxygen, 6-minute walk test, SBP – systolic blood pressure, DBP – diastolic blood pressure, CAT - COPD Assessment Test, FVC – forced vital capacity, FEV1 – forced expiratory volume in 1 second, MEF - maximum expiratory flow rate (MEF), LV – left ventricle, LA – left atrium, RA – right atrium, RV – right ventricle, EDV - end diastolic volume, ESV - end systolic volume, SV – stroke volume, eSPPA - estimated systolic pressure in the pulmonary artery, TAPSE - Tricuspid annular plane systolic excursion, pCO_2 - partial pressure of carbon dioxide, pO_2 - partial pressure of oxygen.
